# Sub-genomic analysis of Chikungunya virus E2 mutations in Pakistani isolates potentially modulating B-cell & T-Cell immune response

**DOI:** 10.12669/pjms.37.1.3236

**Published:** 2021

**Authors:** Bilal Ahmed Khan, Saifullah, Amanullah Lail, Saeed Khan

**Affiliations:** 1 Bilal Ahmed Khan, M.Phil. Department of Biotechnology, University of Karachi, Karachi, Pakistan, Department of Pathology, Dow University of Health Sciences, Karachi, Pakistan; 2 Dr. Saifullah, Ph.D. Department of Biotechnology, University of Karachi, Karachi, Pakistan; 3 Dr. Amanullah Lail, FCPS. Department of Pediatrics, Dow University of Health Sciences, Karachi, Pakistan; 4 Prof. Dr. Saeed Khan, Ph.D. Department of Pathology, Dow University of Health Sciences, Karachi, Pakistan

**Keywords:** CHIKV, E2 Mutation, Epitope, Pakistan

## Abstract

**Background & Objectives:**

The Chikungunya virus (CHIKV) transmitted to the humans through *Aedes* species of the mosquitoes. In December 2016, a severe outbreak reported from Pakistan. However, there is no vaccine or anti-viral treatment currently available so host immune response against CHIKV gained significant interest. Therefore, this study was conducted to identify the mutations in CHIKV E2 region of currently circulating Pakistani strains & determine their potential immunogenicity in Pakistani population.

**Methods:**

It was a cross sectional study in which a total of 60 CHIKV PCR positive samples were collected from Molecular Department of Pathology, Dow University of Health Sciences (DUHS), Karachi during November 2017 to February 2018. CHIKV E2 gene was amplified by PCR & sequenced. Sequences were analyzed by using bioinformatic tools followed by epitope prediction in E2 sequences by *In-silico* immunoinformatic approach.

**Results:**

Several single nucleotide variations (SNVs) were identified in Pakistani isolates with six novel mutations in E2 sequences. Immunoinformatic analyses showed more proteasomal sites, CTL & B-Cell epitopes in Pakistani strains with respect to S27 prototype with 69.4% population coverage against these epitopes in Pakistan. The study also identified key mutations responsible for generation of unique epitopes and HLA restriction in Pakistani isolates. The strain specific mutations revealed the current outbreak was caused by ESCA.IOL lineage of CHIKV.

**Conclusion:**

The evolution of E2 protein in Pakistani strains has increased its immunogenicity in comparison to ancestral s27 strain. The identification of most immunogenic and conserved epitopes with high population coverage has high potential to be used in vaccine development against these local strains.

## INTRODUCTION

CHIKV is single stranded positive sense RNA arbovirus which is transmitted through *Aedes* *spp*. of mosquitoes. It was first isolated from Tanzania in 1953.^1^ Approximately 50%-97% of patients develop symptoms including acute fever and persistent arthralgia in the chronic phase. The ~11.8 kb genome of CHIKV encodes for three structural proteins (E1-E3), core protein and four non-structural proteins (NSP1-NSP4). Infection is usually self-limiting but in some patients severe joints pain can persist for several years.^2^ Some studies also suspected its involvement in development of neonatal encephalopathies, fulminant hepatitis & neurological disorders.^3^

Before 2000, sporadically outbreaks of naturally acquired infections were reported from many African countries.^4^ Since 2000, the virus has re-emerged & caused severe epidemics of unequalled magnitude with more severe form of disease & further spread to other American regions.^5^,^6^ In December 2016, a severe outbreak for the first time was officially reported to the WHO from the Malir district of Karachi Pakistan. More than 30,000 peoples were infected, based on the clinical investigations of which 4000 cases were already confirmed through qualitative RT-PCR.^7^

Currently there is no approved treatment or vaccine available against CHIKV infection. The viral clearance is dependent upon the immunological response, which recognizes the epitopes of viral antigens.^8^ Previous studies have showed that E2 is the most immunogenic part of CHIKV proteome. It is also important for the induction of early immune response in acute phase & produce long lasting post-infection immunity.^9^ However, different studies have identified several epitopes spanning the entire E2 protein but these epitopes evolved as viral strains acquire different mutations in each population during the outbreak and the immune response also varies due to human leukocyte antigen (HLA) types coverage in each population.^10^ Therefore, this study was conducted to identify the mutations in CHIKV E2 region of currently circulating Pakistani isolates & determine their potential immunogenicity in Pakistani population.

## METHODS

It was a cross sectional study approved from Institutional Review Board of DUHS (Ref: IRB-1210/DUHS/Approval/2019, Dated:19/02/19) total of 60 CHIKV positive serum samples were collected from Molecular Pathology Section DUHS during November 2017 to February 2018. Viral RNA was extracted using QIAamp Viral RNA Mini Kit (Qiagen, Germany) and reverse transcribed in to cDNA. Which was used in PCR reaction to amplify the E2 gene with forward primer E2-F 5`AGCACCAAGGACAACTTCAAT-3` & reverse primer E2-R 5`TTTAGCTGTTCTGATGCAGC-3` using DreamTaq Green PCR Master Mix (ThermoScientific, U.S.). The amplified products were run on 1% Agarose and sent for commercial DNA sequencing after purification to Macrogen, Korea.

The generated sequences aligned against the CHIKV-S27 strain (AF369024.2) and first published CHIKV-Pakistani isolated (MF740874) using MEGA7.These sequences were *In-silico* translated in to amino-acid sequences by using Expasy. To predict and compare the immunogenicity of CHIKV E2 gene of Pakistani isolates with S27 ancestral strain different immunoinformatic tools were used. Briefly, NetChop3.1 was utilized for the prediction of proteasomal degradation sites with 0.5 threshold.^11^ CD8+T cell (CTL) epitopes with their restricting HLAs were predicted by CTLPred & nHLAPred respectively.^12^ The population coverage for MHC-I binders for these epitopes were predicted by IEDB population coverage tool.^13^ B-cells linear epitopes were predicted by using IEDB-BEpipred prediction tool.^14^

## RESULTS

**Fig.1:**
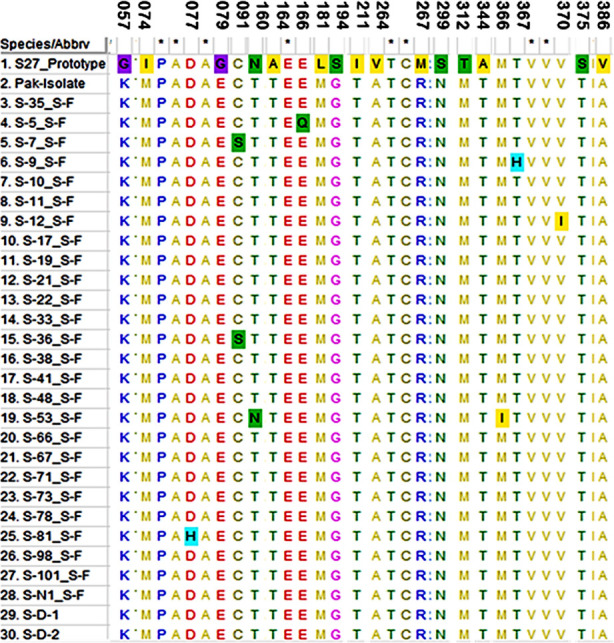
Mutation Analyses of CHIKV E2 Amino Acid Sequences. Each SNV are highlighted with color and positions are marked above the alignment.

Out of 60 positive patients, 34 (56.6%) were males & 26 (43.4%) were females while the mean age of patients were 33.5 years. The E2-gene was successfully amplified & sequenced from 30 patients only as other samples were not amplified possibly due to low viral load in the sample. The mutation analyses revealed 21 different mutations in CHIKV-E2 amino acid sequences among which 15 mutations were already observed in CHIKV-Pakistani isolate sequenced previously from early outbreak. Six novel mutations including C91S in two and D77H, E166Q, M366I, T367H, V370I is present in one sample respectively from this study only (Fig.1).

**Fig.2:**
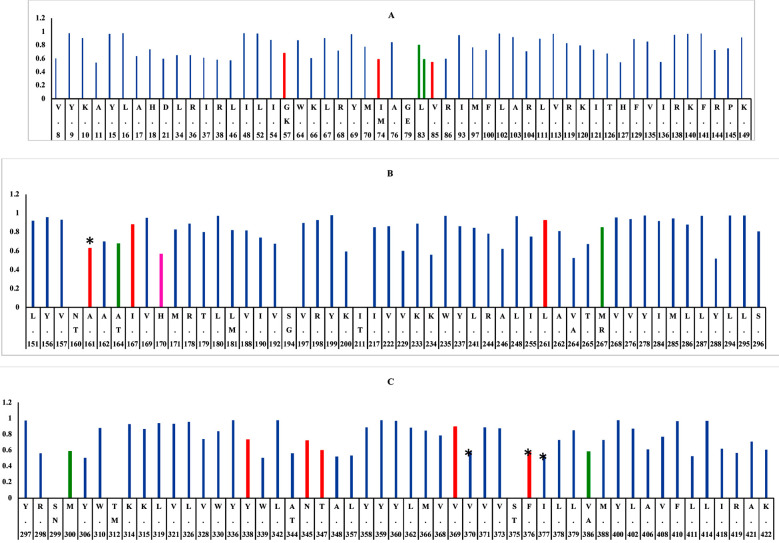
Proteasomal cleavage sites in CHIKV-E2. Blue color indicate site common for both, Red indicate site only present in S27 strain, Green indicate site only present in Pakistani strains. Pink color showed additional site in one Pakistani strain due to SNV while asterisk (*) indicates deletion of these sites in some Pakistani strains due to SNVs.

Comparative analyses of proteasomal degradation sites revealed that E2 sequences from Pakistani isolates & this study have 141.9±0.6 proteasomal degradation sites in comparison with 135 sites in S27-prototype sequence. Unique proteasomal degradation sites were observed on position 57, 74, 85, 86, 161, 167, 262, 339, 347, 348, 370, 376, 377 while five sites on position 83, 164, 267, 300, 386 were obliterated but were present in s27-prototype strain (Fig.2). The gain/loss of sites were due to the acquisition of certain mutations in E2 region. Briefly mutations of C91S, N160T & E166Q created new sites on position no 83, 161 & 160 respectively in four samples of this study. In-contrary mutation of V370I obliterated three sites on position 370,376 & 377 in one sample.

**Fig.3:**
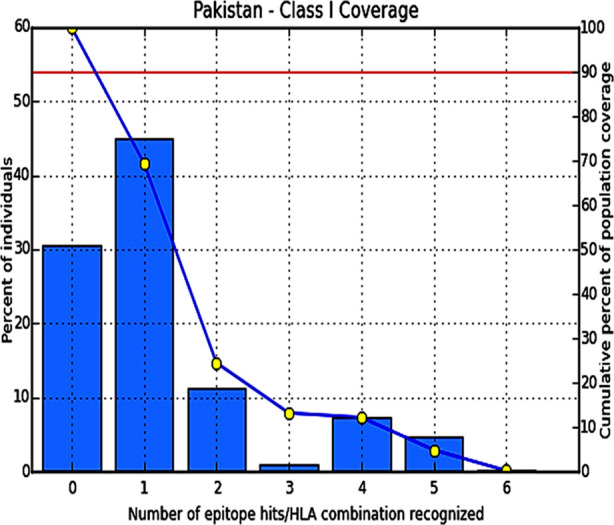
Population Coverage for CHIKV E2 epitopes in Pakistani population.

To further elucidate the immunogenic properties of CHIKV-E2, CTL epitopes with restricting class-I HLAs were predicted by using CTLPred and nHLAPred tools respectively. The results showed that both groups have 15 epitopes however samples from this study have five unique epitopes including the three newly evolved epitopes & two modified epitopes due to point mutations. While s27-E2 sequence revealed one absolutely unique epitope which has been obliterated in Pakistani strains. The mutations resulted in intra-epitope variability also effected its HLA class-I restriction (Table-I). Briefly, SNV S375T in E2 sequences from Pakistani isolate allowed it to be restricted by HLA-B*5102 and HLA-B*5103 in addition to HLA-Cw*0401.Similarly, SNVs V264A and M267R also created new HLA-A24 restriction site in addition to HLA-A*0301 & HLA-Cw*0401 in S27-Prototype strain. Pakistani population coverage for MHC-I binders for these epitopes revealed 69.4% population coverage for these epitopes in Pakistani population (Fig.3).

**Table-I:** CTL epitopes in CHIKV-E2 sequences.

Start Position	Epitope	ANN/SVM Score	Restricting HLA
1	STKDNFNVY	0.98/1.05	HLA-A*0206, HLA-A3, HLA-B*51,HLA-Cw*0401
143	SRPQHGKEL	0.95/1.02	HLA-Cw*0401
371	VSVA[T/S]FILL	0.95/0.90	HLA-B*5102, HLA-B*5103,HLA-Cw*0401
271	ARNPTVTYG	0.88/0.87	HLA-Cw*0401
363	YPTMTVVVV	0.86/0.84	HLA-B*5301, HLA-B*51, HLA-Cw*0401
	YPTMHVVVV	0.86/0.54	HLA-B*51, HLA-Cw*0401
	YPTMTVVIV	0.86/1.39	HLA-A*0301, HLA-B*5301, HLA-Cw*0401
	YPTITVVVV	0.86/0.91	HLA-B*5301, HLA-B*51, HLA-Cw*0401
365	TMTVVVVSV	0.68/0.96	HLA-B*5301, HLA-B*51, HLA-Cw*0401
276	VTYGKNQVI	0.99/0.63	HLA-A11, HLA-A3, HLA-Cw*0401
221	KVDQCHAAV	0.55/1.07	HLA-B*51, HLA-Cw*0401
130	HHDPPVIGR	0.95/0.42	HLA-Cw*0401
78	AERAGLFVR	0.72/0.62	HLA-Cw*0401
313	HKKEVVLTV	0.82/0.47	HLA-A*0301, HLA-B*5301, HLA-Cw*0401
270	KARNPTVTY	0.89/0.38	HLA-A1, HLA-B*51, HLA-Cw*0401
260	PLAN[A/V]TC[R/M]V	0.67/0.51	HLA-A24, HLA-A*0301, HLA-Cw*0401
368	VVVVSVATF	0.66/0.42	HLA-Cw*0401
212	TTDKVINNC	0.54/0.52	HLA-Cw*0401
385	AVGMCMCAR	0.98/0.37	HLA-Cw*0401
211	ITTDKVINN	0.91/0.39	HLA-Cw*0401
373	VASFILLSM	0.58/0.39	HLA-Cw*0401
159	STAATTEQI	0.81/0.42	HLA-A11, HLA-A3, HLA-A31, HLA-Cw*0401

Epitopes specific for s27 and Pakistani isolates are marked red and blue respectively. Epitopes variation are marked with green Epitopes specific for s27 and Pakistani strains are marked red and blue respectively.

To predict the potential antibody response, B-cell linear epitopes revealed 14 potential epitopes in comparison to 13 epitopes in s27-strain. Generation of one new epitope and intra-epitope variability in Pakistani sequences were the result of evolutionary acquisition of mutations in these strains over the years (Table-II).

**Table-II:** B-Cell Epitopes in CHIKV-E2 Sequences.

Position	Epitope	Length
6-17	FNVYKATRPYLA	12
20-30	PDCGEGHSCHS	11
36-42	RIRNEAT	7
56-80	IKTDDSHDWTKLRYMDNHMPADAER	24/25
118-121	SRKI	4
131-165/177	HDPPVIGREKFHSRPQHGKELPCSTYVQS[T/N]AAT[T/A]EEIEVHMPPDTPD	35/47
172-178	PPDTPDR	7
207-212/210	NEGLTT	6/3
215	K	1
217/218-219	INN	3/2
234-252	KWQYNSPLVPRNAELGDRK	19
273-278	NPTVTY	6
299/300-314	NMGEEPNYQEEWV[M/T]HK	16/15
330-350	WGNNEPYKYWPQLS[T/A]NGTAHG	21
397-398	IT	2

Epitopes specific for s27 strain are marked red, while epitope specific for Pakistani strains are marked blue.

## DISCUSSION

CHIKV epidemic with evolution of novel mutations are the major global health. Mutations in the E1 protein has been proven to be the main reason for the epidemic potential of CHIKV re-emergence due to altered vector specificity and infectivity.^15^ Several second step mutations in E2 have been document that alter the fitness of CHIKV in mosquito vector.^16^,^17^ In this study, *Ae. albopictus* adaptive mutation K252Q were not observed although it was reported from 8 CHIKV isolates sequenced from early outbreak in Pakistan.^18^ Instead V264A were consistently present in all samples which enhances the fitness of CHIKV in *Ae.aegypti*.^19^ This speculates some involvement of *Ae. albopictus* in the early outbreak and major contribution of *Ae. aegypti* as a main vector for CHIKV transmission in Pakistan. Most of the mutations observed in E2 were previously reported in ESCA strains around the world including eight Pakistani sequences from the early outbreak. Specifically, V386A & I211T were also observed in this study which is most frequently present in ECSA.IOL lineage thus concluding the current CHIKV outbreak in Pakistan was caused by CHIKV-ESCA.IOL lineage. We also observed six novel mutations in E2 which is known to be the most variable protein of CHIKV thus indicating further viral evolution in the community due to selective pressure.^20^

This study also predicted the CHIKV-E2 antigen processing and presentation by *In-silico* approach. Cellular proteasome chops the antigens at preferred sites then these peptides bind with Class-I HLA and presented on cellular surface of APC where CTLs recognize them and elicit immune response. Mutations in up or down stream of epitopes can alter CTL response which may result in impaired protection against CHIKV infection or vice versa.^21^,^22^ The evolutionary acquisition of mutations in CHIKV-E2 generated unique proteasomal cleavage sites (n=142 sites) in current CHIKV-Pakistani strains in comparison with ancestral S27-Strain (n=135 sites). The novel mutations created one new site while obliterated three sites which are not reported in early outbreak from Pakistan. However, increase in number of proteasomal sites in current strains indicates more effective antigen processing for presentation in comparison to ancestral strains.^23^

The influence of these mutations on the generation of CTL epitopes showed three new epitopes & obliteration of one epitope in Pakistani strains. The intra-epitope variation also affected the HLA restriction of these epitopes. SNV S375T in epitope VSVASFILL of S27 allowed it to be restricted by two additional HLAs in current Pakistani strains. Similarly, SNVs V264A and M267R also generated additional MHC-I binding site (Table-I). The epitopes STKDNFNVY & SRPQHGKEL are found to be the most immunogenic in both strains indicating highly conserved nature of these sites throughout viral evolution. The currently circulating Pakistani strains also have one additional B-cell epitope and intra-epitope variability in others. The generation of epitope and variability on B-cell epitope on position 207 are most probably due to the mutation I211T while epitope variability on position 131 is also supposedly due to N160T and A164T as mutations in epitope regions are known to alter the immunogenicity of viruses 24,25.Moreover, population coverage analysis also showed that these epitopes have 69.4% population coverage in Pakistani population which has never been evaluated before for CHIKV. This might be one of the possible reasons that the CHIKV outbreak was not sustained for longer period in Pakistan. Moreover, sequencing of only E2-gene rather than all coding region limited the potential immunogenicity prediction against this region only which should be further evaluated for other structural proteins.

## CONCLUSION

The mutations acquired in E2 protein of Pakistani strains have increased immunogenicity in comparison to ancestral s27-strain. Moreover, the identification of most immunogenic and conserved epitopes with high population coverage have the high potential to be use in vaccine development against these local strains.

### Authors’ Contribution:

**BAK** performed the experimental work & manuscript writing.

**SU** did the data analysis & manuscript editing.

**AL** did the sample, data collection and reviewed the manuscript.

**SK** conceived, designed the study & did bioinformatics analyses. He is also responsible for integrity of work.
